# An analysis of the use of genomic DNA as a universal reference in two channel DNA microarrays

**DOI:** 10.1186/1471-2164-6-66

**Published:** 2005-05-08

**Authors:** Mugdha Gadgil, Wei Lian, Chetan Gadgil, Vivek Kapur, Wei-Shou Hu

**Affiliations:** 1 Department of Chemical Engineering and Materials Science, University of Minnesota, 421 Washnigton Ave. S.E., Minneapolis, MN 55455 USA; 2 School of Mathematics, University of Minnesota, 270A Vincent Hall, Minneapolis, MN 55455 USA; 3 Scientific Computing and Mathematical Modeling, GlaxoSmithKline, Research Triangle Park, NC 27709 USA; 4 Department of Microbiology, University of Minnesota, MMC 196, 420 Delaware Street, S.E., Minneapolis, MN 55455 USA

## Abstract

**Background:**

DNA microarray is an invaluable tool for gene expression explorations. In the two-dye microarray, fluorescence intensities of two samples, each labeled with a different dye, are compared after hybridization. To compare a large number of samples, the 'reference design' is widely used, in which all RNA samples are hybridized to a common reference. Genomic DNA is an attractive candidate for use as a universal reference, especially for bacterial systems with a low percentage of non-coding sequences. However, genomic DNA, comprising of both the sense and anti-sense strands, is unlike the single stranded cDNA usually used in microarray hybridizations. The presence of the antisense strand in the 'reference' leads to reactions between complementary labeled strands in solution and may cause the assay result to deviate from true values.

**Results:**

We have developed a mathematical model to predict the validity of using genomic DNA as a reference in the microarray assay. The model predicts that the assay can accurately estimate relative concentrations for a wide range of initial cDNA concentrations. Experimental results of DNA microarray assay using genomic DNA as a reference correlated well to those obtained by a direct hybridization between two cDNA samples. The model predicts that the initial concentrations of labeled genomic DNA strands and immobilized strands, and the hybridization time do not significantly affect the assay performance. At low values of the rate constant for hybridization between immobilized and mobile strands, the assay performance varies with the hybridization time and initial cDNA concentrations. For the case where a microarray with immobilized single strands is used, results from hybridizations using genomic DNA as a reference will correspond to true ratios under all conditions.

**Conclusion:**

Simulation using the mathematical model, and the experimental study presented here show the potential utility of microarray assays using genomic DNA as a reference. We conclude that the use of genomic DNA as reference DNA should greatly facilitate comparative transcriptome analysis.

## Background

The rapid increase in the number of completely sequenced genomes in the past few years has generated much effort in functional genomics, particularly studies seeking to assign biological functions to DNA sequences. Comparative gene expression profiling is widely used to study the functional role of genes. The DNA microarray assay provides an invaluable technique for large scale expression analysis. In the two-channel DNA microarray assay, RNA from two samples is reverse transcribed to cDNA and labeled with two distinct fluorescent dyes before being co-hybridized to immobilized DNA strands on a microarray slide. Spotted arrays currently being used can be divided into two groups based on the nature of immobilized DNA used: one in which the immobilized DNA is comprised of both sense and antisense strands (usually PCR product) and the other where the immobilized DNA is single stranded consisting of only the sense strands (usually, chemically synthesized oligonucleotides). During hybridization, the two fluorescently labeled cDNA samples compete for hybridization to the immobilized strands. Hybridization reactions between complementary strands occur only between the labeled antisense strand and immobilized sense strand. The ratio of the intensities of the two fluorescently labeled cDNAs is used to quantify the relative levels of transcripts in the two samples [1,2]. This method serves well for pair-wise comparison of transcript levels in two samples. With over ten thousand different DNA species immobilized on the microarray, the relative transcription level of all the corresponding genes in the two samples can be obtained in a single assay.

DNA microarrays have found applications in gene discovery, disease diagnosis, pharmacogenomics and toxicology research. They are increasingly used for a series of related samples, for which a comparison across all samples and all genes is desirable. When a large number of samples are to be compared, a combinatorial approach pairing all possible pairs (or at least a number of combinations of pairings of the sample) is often taken. This results in a large number of microarrays, requiring a large amount of each RNA sample. A 'loop design', where every sample is directly compared to two other samples to form a closed loop, has been proposed to overcome this problem [3,4]. The ratios calculated using a loop design have variable levels of precision since some samples are more directly related than others [5]. When a new sample is to be inserted into the earlier 'loop', RNA for at least two of the previous samples is needed to pair with the new sample to form a new node in the closed loop.

Another approach to tackle the issue of a large combinatorial pair-wise comparison is the 'reference design' [3] in which a common reference sample is introduced with which all RNA samples are hybridized. Two possible universal references are RNA pooled from various samples and genomic DNA [6]. For a given set of samples, pooled RNA provides an excellent reference. However, if the experimental conditions change, the possibility arises that some new transcripts may not be represented in the initially-pooled RNA.

Genomic DNA is an attractive candidate for use as a common reference. It is isolated from cells or tissue and sheared to fragments in a narrow range of length. It is easier to prepare, maintain and reproduce, as compared to RNA. It is especially useful for microorganisms, which lack repetitive sequences in their genome, and microarrays using genomic DNA as a reference have been used to identify genes differentially expressed in various growth stages of *Mycobacterium tuberculosis *[7]. It has also been recently reported that the data obtained using genomic DNA as a reference in microarray experiments with *Arabidopsis thaliana *employing 70-mer oligonucleotide microarrays was in agreement with ratios obtained from direct hybridizations [8]. Genomic DNA samples, isolated from stationary phase cultures where the chromosome is not being replicated, have the same representation of all genes as in the genome. Since the transcript level of each gene is being referenced to its own representation on the genome (for most genes, it is a single copy), the relative expression can be compared across different genes in the sample (i.e., from the same hybridization) as well as across different samples. The use of microarrays using genomic DNA in a range of applications including genomic diversity studies [9,10] and aneuploidy detection using comparative genomic hybridization [11] has been demonstrated.

In the conventional two-channel cDNA hybridization [1], both the cDNA samples are antisense strands. The probability of hybridization between strands in solution is very low. On the other hand, using sheared genomic DNA as a reference, hybridization between complementary sense and anti-sense strands can occur in solution between the complementary genomic DNA strands, and between genomic DNA sense strands and their cDNA counterparts. The number of strands lost to hybridization in the solution phase may differ for different RNA species as well as for the two complementary strands of the same species. This may result in decreased fidelity in the ratio of cDNA to genomic DNA as a representation of gene expression level. With the complexity of hybridization in both the solution phase and the immobilized surface phase, and between double strands of genomic DNA and single strands of cDNA, it is difficult to assess the effect of using genomic DNA as a common reference. Adapting a mathematical model we developed previously to assess diffusional constraints on DNA microarray assay [12], we have constructed a kinetic model for microarray hybridization and predicted the effectiveness as well as potential pitfalls of using genomic DNA as a reference. We examined the effect of the various parameters that may affect surface hybridization. Here we report the framework of the model and our findings.

### Mathematical model for microarray hybridization

**Figure 1:**
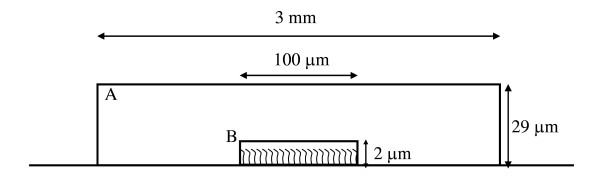
Schematic of the system for simulating hybridization for a two-color assay. A: Solution phase, B: spot phase

A DNA microarray consists of thousands of spots, each spot containing DNA strands of known sequence immobilized on an impermeable surface. We simulate hybridization reactions for one spot on the microarray, which is considered to be a sample chamber with two compartments; a spot phase and a solution phase (Figure 1). The spot phase is the small volume in which the bound strands are assumed to be present at a uniform concentration. The solution phase, which comprises a vast majority of the volume of the microarray chamber, contains only fluorescently labeled single strands at the beginning of the hybridization and double strands formed by hybridization of complementary mobile single strands during the course of the hybridization.

Five kinds of single-stranded DNA molecules are present in the system: labeled single stranded cDNA (**S**ample) reverse transcribed from RNA, denoted as S; the two identically labeled complementary (anti-sense and sense) strands from genomic DNA (**R**eference), denoted as R and R'; and the non-labeled anti-sense and sense strands immobilized on the array surface (**B**ound strands) denoted by B and B'. The nomenclature used in this study is summarised in Table 1. The. The double-stranded species are denoted by combining the constituent single strand symbols, for example RB' denotes the labeled complex formed by hybridization of genomic DNA antisense strands to the sense bound strands. We assume that each phase is well-mixed, and hence all mobile species are present at uniform concentrations within each phase.

**Table 1:** Nomenclature

**Symbol**	**Description**
S	cDNA sample anti-sense strand
R	Genomic DNA anti-sense strand
R'	Genomic DNA sense strand
B	Bound (immobilized) anti-sense strand
B'	Bound (immobilized) sense strand
Subscript 0	Initial concentration
SR'	Double strand formed in solution by hybridization of anti-sense cDNA strand and sense genomic DNA strand
RR'	Double strand formed in solution by hybridization of anti-sense and sense genomic DNA strands
SB'	Double strand formed on surface by hybridization of anti-sense cDNA strand and sense bound strands
RB'	Double strand formed on surface by hybridization of anti-sense genomic DNA strand and sense bound strands
R'B	Double strand formed on surface by hybridization of sense genomic DNA strand and anti-sense bound strands
h	Height of spot phase
r	Radius of spot phase
k_b_	Rate constant of backward reaction of hybridization
k_f_	Rate constant of forward reaction of hybridization between mobile species
k_f-bound_	Rate constant of forward reaction of hybridization between mobile and bound species
k_t_	Rate constant for transport between the two phases
γ	Ratio obtained from a hybridization assay using genomic DNA as a reference { = [SB']/([RB']+[R'B])}
α	Assay efficiency = (γ_1_/γ_2_)/(S_10_/S_20_), where 1 and 2 denote samples 1 and 2
ε_S_	Amount of S reacted with R'
ε_R_	Amount of R reacted with R'

We consider the case that PCR products are used for immobilization; thus, both sense and antisense strands of probe DNA are immobilized. We assume that the two complementary bound strands do not hybridize to each other [13] due to the immobilization procedure used and neglect the formation of BB'. The mobile species in the two compartments are considered to move across the phase boundary at a rate proportional to the difference in the concentrations of identical species present in the two phases. The proportionality constant is the effective mass transfer coefficient for the transport of mobile DNA strands and is estimated from the diffusivity as discussed below.

The model equations take the form of a mass balance on each component in each phase that accounts for the change in concentration due to reactions within that phase and transport between the two phases. Non-specific hybridization in both phases is neglected. All these equations are of the form:



where  = R',  = B', A_1 _= R, A_2 _= B, A_3 _= S, subscript p denotes either solution or spot phase and p' is the other phase; k_f _is the forward rate of hybridization; k_b _is the backward rate of hybridization; k_t _is the rate of transport across the spot-phase solution-phase boundary; V_t _is the total volume of the sample chamber and V_p _is the volume of phase p.

### Model parameters

#### Model geometry

The diameter of the spot phase is set to 0.01 cm as seen in a typical microarray spotted on poly-lysine slides [14], with a height of 2 × 10^-4 ^cm (Figure 1). This gives a spot volume of 0.2 × 10^-12 ^l. Typically, labeled strands are resuspended in a volume of 4.5 × 10^-5 ^l, and this volume is applied under a cover slip of area 6.5 cm × 2.4 cm. For this geometry, the height of the microarray chamber is about 2.9 × 10^-3 ^cm. The solution phase volume is the difference between the sample chamber volume (4.5 × 10^-5 ^l) and the spot volume (0.2 × 10^-12 ^l). It has been shown before that only the mobile strands within a radius of 0.15 cm from the spot are available for hybridization due to transport effects [12]. Hence, we have assumed the solution phase to have a diameter of 0.15 cm and a height of 2.9 × 10^-3 ^cm corresponding to the height of the microarray chamber.

#### Hybridization rate constants

The hybridization rate constants are assumed to be identical for DNA strands from both genomic DNA and cDNA. However, the rate constants for reaction between two mobile strands may differ from the rate constant for the reaction of a mobile strand and a bound strand. A forward rate constant (k_f_) for DNA hybridizations in solution of 10^6 ^M^-1 ^s^-1 ^[15,16] was used for simulations. The rate constant of hybridization could be slower for hybridization of mobile strands to immobilized strands (k_f-bound_) on a solid surface and values in a range up to 100-fold lower than k_f _have been used in simulations. We also discuss the effect of this reduced forward rate constant of hybridization between mobile and bound strands for a range between 10^6 ^M^-1^s^-1 ^and 10^4 ^M^-1^s^-1^. The backward rate constant for dissociation of DNA double strands as calculated from equilibrium constants reported in literature ranges from 10^-3 ^s^-1 ^to 10^-1 ^s^-1 ^[17,18]. Simulations were carried out using a backward rate constant ranging from 10^-1 ^s^-1 ^to 0 s^-1 ^(irreversible). In all simulations performed, the deviation from the true value was greatest when the hybridization reaction is set to be irreversible. The results discussed below are for the case with the backward rate constant set to 0 s^-1^. The results obtained when the reaction is set to be reversible are very close to the true value under all conditions tested.

#### Rate constant for transport

The rate constant for transport (k_t_) is estimated from the diffusion coefficient as , where D is the diffusion coefficient, h is the height of the spot phase and r is the radius of the spot phase. The diffusivity of DNA single strands in solution has been estimated to be 10^-7 ^cm^2^/s [19-21] which leads to an estimate of 1 s^-1 ^for k_t_. The simulations reported in the next section were carried out under both very fast transport (k_t _= 10^-3 ^s^-1^) and very slow transport (k_t _= 10^-3 ^s^-1^) conditions. The transport rate has no effect on the performance of the assay in the range tested (data not shown).

#### Initial single-strand concentrations

From the yeast transcriptome data published by Velculescu *et al*. [22], the mass percentages of mRNA that belong to the rare, intermediate and abundant classes are estimated to be 65.2, 32.4 and 2.4% respectively. For an mRNA sample of 0.2 μg used in each microarray assay, the total amount of rare, intermediate and abundant species are 0.13 μg, 0.07 μg and 4.8 × 10^-3 ^μg respectively. To convert those numbers to molar concentration we calculate the number of genes within each abundance class using the intensity data from microarray experiments of *S. coelicolor*. The *S. coelicolor *transcripts were divided into three abundance classes using the following intensity cut-offs: Intensity < 2000 as rare, 2000 < Intensity < 20000 as intermediate and >20000 as abundant sequences. Normalized intensity values were used for this estimate. 72.8%, 26% and 1.2% of all genes were classified rare, intermediate and abundant respectively. The corresponding number of rare, intermediate and abundant species are 5697, 2034 and 94 respectively. Assuming a sample volume of 4.5 × 10^-5 ^l, 100% reverse transcription efficiency, and an average strand length of 1000 we calculate the rare species concentration as ~0.1 pM, intermediate species concentration ~1 pM and abundant species concentration ~20 pM.

Previous experimental reports of the use of genomic DNA as a reference for microarray hybridizations used genomic DNA concentrations ranging from 0.1 μg to 4 μg for *M. tuberculosis *[7]. Since *M. tuberculosis *has ~4000 genes, this translates to a concentration of each gene from ~1 pM to 34 pM, which is the range of genomic DNA concentrations used for simulations.

To calculate the concentration of the immobilized species, we assume the concentration of DNA in the spotting solution to be 0.1 g/l and that 10^-9 ^l of the solution is spotted on the microarray. We also assume that 75% of the DNA thus spotted is washed away in the microarray post-processing steps. 2.5 × 10^-11 ^g DNA remaining on the microarray is uniformly distributed in the 0.2 × 10^-10 ^l spot phase volume. Assuming an average DNA strand length of 1000 bp, this is approximately equal to an immobilized strand concentration in the spot phase of 10^-6 ^M.

## Results and Discussion

The mathematical model described above takes into account DNA hybridization between single stranded cDNA and double stranded genomic DNA (gDNA) in solution and immobilized double strands on a microarray surface. This model considers hybridization only on one spot on the microarray. The immobilized strands are distributed uniformly in the spot phase and the mobile strands are present both in the solution phase and spot phase. Hybridization between mobile and bound species occurs in the spot phase. All concentrations described in the following sections are the concentrations in the spot phase. The fluorescence intensity corresponding to hybridized cDNA sample is expressed as I_S _= [SB'] and the channel corresponding to the hybridized genomic DNA reference as I_R _= [RB'] + [R'B]. The result obtained for hybridization with genomic DNA used as a reference is a hybridization ratio γ = I_s_/I_R _= [SB'] / ([RB'] + [R'B]). Since the concentration of all genes in a genomic DNA sample is equal, the ratio (γ) for different genes is an indication of the relative abundance of the transcript for those genes.

Typically when genomic DNA is used as the reference, the ratio (γ_1_) from one hybridization of cDNA derived from sample one, is compared to another ratio (γ_2_) from sample two, to obtain the relative expression level of the transcripts in samples 1 and 2. Ideally this "ratio of ratios", i.e. γ_1_/γ_2_, should be equal to the ratio of the initial concentrations of the transcript in those samples. We simulate this experimental process with two different initial cDNA concentrations (S_10 _and S_20_) and the same genomic DNA concentration to obtain the ratios γ_1 _and γ_2_. The accuracy of the microarray assay is quantified by the accuracy index α,



A value of one for α corresponds to a perfect assay, where the measured relative concentration of the transcript in the two samples (γ_1_/γ_2_) is exactly equal to the true relative concentration (S_10_/S_20_). Any deviation in α from unity is a measure of the error of the assay.

These results are also applicable to the comparison of expression levels of two genes in one cDNA sample, as the model makes no distinction between hybridization on two spots on one microarray and hybridization to a spot corresponding to the same sequence in two different microarray experiments. We systematically vary model parameters to investigate the effect of different hybridization conditions, transcript abundance levels, and degree of differential expression on the performance of the microarray assay.

### Effectiveness of using genomic DNA as a reference

**Figure 2:**
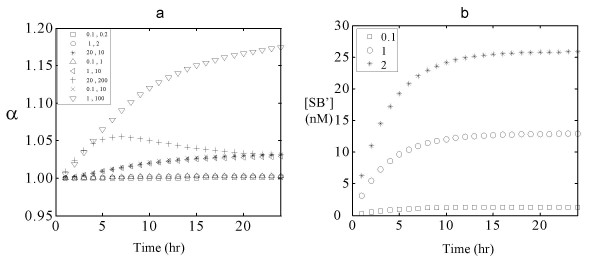
Effect of hybridization time on α and [SB'] for different levels of RNA abundance and differential expression. 2a) The variation in α with the hybridization time is shown for 8 different cases of abundance level and differential expression listed in Table 2. The parameters used are k_f _= 10^6 ^M^-1^s^-1^, k_f-bound _= 10^6 ^M^-1^s^-1^, k_b _= 0 s^-1^, initial genomic DNA concentration = 1 pM, bound strand concentration = 10^6 ^pM, transport rate = 1 s^-1 ^2b) Change in concentration of SB', the intensity corresponding to the cDNA channel, with time for rare and intermediate species. □ S_0 _= 0.1 pM, ○ S_0 _= 1 pM, * S_0 _= 2 pM

The effectiveness of using genomic DNA as a reference in the microarray assay was predicted by simulations using the model and parameters described above. Figure 2a shows the variation in α with hybridization time for different RNA abundance levels and differential expression ratios corresponding to biologically realistic scenarios as listed in Table 2. These cases represent a wide range of possible combinations of the three abundance classes and differential expression ratios (2, 10 and 100). A differential expression ratio of 2 is used as an example of a small change in expression level and 100 as an example of a large change in expression level. The parameter values used are k_f _= 10^6 ^M^-1^s^-1^, k_f-bound _= 10^6 ^M^-1^s^-1^, k_b _= 0 s^-1^, initial genomic DNA concentration = 1 pM, initial bound strand concentration = 10^-6 ^M, k_t _= 1 s^-1^. For a wide range of initial cDNA concentrations and differential expression ratios, the accuracy index α is within 5% from unity (Figure 2a), indicating that the assay performance is robust to the initial concentration of single strands. The only condition where α is significantly different from 1 (~1.17) is when intermediate species are upregulated 100 fold, a situation not likely to happen frequently in cells. Furthermore, in most microarray assays, a 17% deviation from the true value is not considered large. The model simulation predicts that for abundant species, the accuracy decreases with hybridization time, indicating short hybridization time will lead to better results. However, the concentrations of the double strands formed as a result of hybridization of single strands in solution to the immobilized strands (shown in Figure 2b for [SB'] as a function of hybridization time) increase with time for approximately the first 15 hours. The fluorescent intensities detected when the microarray slide is scanned is proportional to the concentration of these double stranded species. Hence the hybridization time has to be long enough to obtain intensities sufficiently above background levels for accurate measurement.

**Table 2:** List of the combinations of abundance levels and differential expression ratios corresponding to biologically realistic scenarios used in simulations

**C_1 _(pM)**	**C_2 _(pM)**	**Differential expression**	**Comment**
0.1	0.2	2	Rare species upregulated 2 fold
1	2	2	Intermediate species upregulated 2 fold
20	10	2	Abundant species downregulated 2 fold
0.1	1	10	Rare species upregulated 10 fold
1	10	10	Intermediate species upregulated 10 fold
20	200	10	Abundant species upregulated 10 fold
0.1	10	100	Rare species upregulated 100 fold
1	100	100	Intermediate species upregulated 100 fold

**Figure 3:**
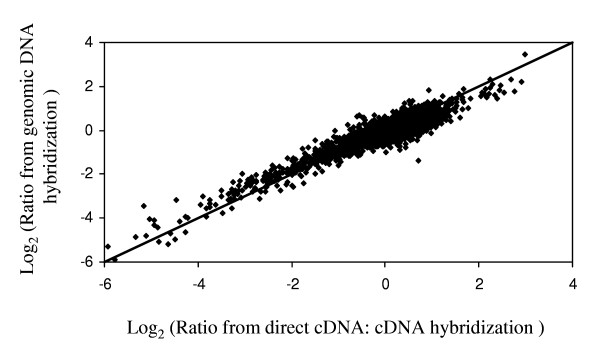
Scatter plot showing log_2 _transformed ratios obtained from direct cDNA: cDNA hybridization and indirect comparison using genomic DNA as a reference. cDNA: cDNA hybridization was carried out using two RNA samples isolated from *S. coelicolor *mycelia obtained from liquid culture at early (Sample 1) and late (Sample 2) growth stages. For the indirect comparison using genomic DNA, each of the two samples was hybridized with genomic DNA.

The model prediction that using genomic DNA as a reference can provide an accurate measurement in a microarray assay was verified experimentally. The transcript levels of two samples were assayed using both direct cDNA: cDNA hybridization (cDNA_1_:cDNA_2_) as well as by hybridizing to genomic DNA (cDNA:gDNA). This ratio of the two samples (γ_1_/γ_2_) was obtained by dividing the ratios obtained from those two cDNA samples ([cDNA_1_/gDNA])/ ([cDNA_2_/gDNA]) (Additional file 1) Microarray data can be found in the Supplementary material. Figure 3 shows a scatter plot of the relative transcript level obtained by these two methods. The ratio obtained from indirect comparison is within 1.5 fold of that obtained from direct comparison for 91% of genes. Out of the remaining 9% genes, 81% have a ratio obtained from direct comparison within 1.5-fold. For 99.3% of all the genes, the ratio obtained from indirect comparison is within 2 fold of that obtained from direct comparison. 70% of the remaining 0.7% have a ratio obtained from direct comparison within 1.5-fold. Thus, in general, the ratio obtained from hybridizations using genomic DNA as a reference is consistent with those obtained from direct cDNA: cDNA hybridization. As can be seen in Figure 3, this is true over a large range of differential expression (128-fold). Also, since total RNA samples containing a wide range of transcript abundance levels were used in this experiment, the dataset demonstrates that the accuracy of the assay is maintained over all mRNA expression levels.

### Effect of genomic DNA concentration

In previous investigations several concentrations of the genomic DNA ranging from 1 to 35 pM have been used [7,23]. To examine the effect of genomic DNA concentrations on the microarray assay, we carried out simulations for a range of genomic DNA (1 to 35 pM) and cDNA concentrations (0.1 to 20 pM) with different degrees of downregulation (2, 10 and 100-fold). α is within 6% of unity for all values of genomic DNA concentrations examined (data not shown). Thus, the assessment of the ratio between the transcript levels of a gene in two samples does not vary significantly with the genomic DNA concentration used in the experiment.

However, in a microarray experiment, accurate assessment of the ratio of expression levels can be attained only if the fluorescent intensities can be accurately measured. Spots with lower intensities and closer to the background level are prone to increased noise interference and decreased accuracy. At higher concentration of genomic DNA, the intensity of the sample channel decreases. This effect is more profound for rare species, compounding the problem of their low intensity. This leads to the need to carefully select the genomic DNA concentration for achieving intensities which are significantly above background levels.

### Effect of bound strand concentration

In the preparation of microarray slides the amount of DNA immobilized on the slide varies. Many factors, including differences in the amount of DNA deposited, spot morphology, DNA retention in the spotting and post-processing procedure contribute to such variation. In cDNA:cDNA hybridization, the ratio of transcript levels from two different samples is not affected by the amount of DNA immobilized on the slides, since the same transcript species from both samples are affected to the same extent. In contrast, when cDNA:gDNA is used, variation in the amount of immobilized DNA may exert a different effect on different samples. We simulated the effect on α of this changing bound strand concentration by using bound strand concentrations of 10^-4 ^and 10^-8 ^M for all cases listed in Table 2. This range spans values from 100-fold higher to 100-fold lower than the estimated bound strand concentration. All other parameters are same as those used for the simulations in Figure 2. The findings are similar to the plot shown in Figure 2a (results not shown). Thus, the variation in the amount of DNA immobilized on slides does not significantly affect the accuracy of the assay. However, a lower bound strand concentration does result in reduced concentrations of the double strands formed, and hence lower intensities on the microarray. As an illustration, when the bound strand concentration is lowered from 10^-6 ^M to 10^-8 ^M, the intensity of the sample channel (corresponding to [SB']) decreases 24-fold for rare species, 25-fold for intermediate species and 38-fold for abundant species.

### Accuracy affected by transcript abundance at low hybridization rate

**Figure 4:**
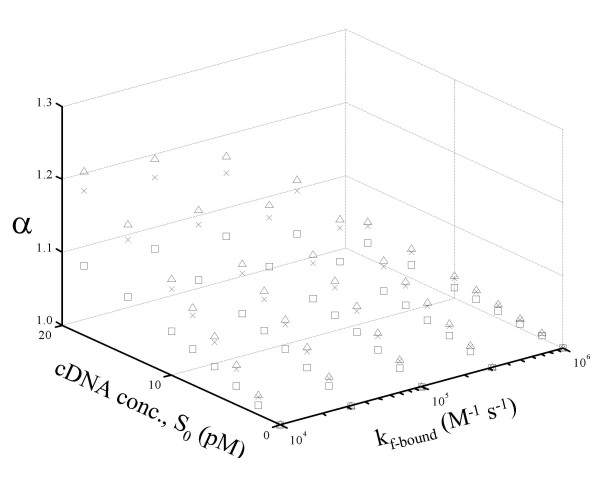
Effect of a decreased rate of hybridization between mobile strands and immobilized strands for different differential expression ratios and RNA abundance levels on α. □ Differential expression = 2 fold, × Differential expression = 10 fold, Δ Differential expression = 100 fold. All other parameters are same as in Figure 2.

An uncertainty in the simulation is the effect of reaction rate constants; especially, the effect of change in the relative magnitude of forward rate constants for hybridization between mobile and mobile, and mobile and bound strands, is further investigated. To evaluate this, k_f-bound _was varied 100-fold from 10^6 ^M^-1^s^-1 ^to 10^4 ^M^-1^s^-1 ^and the results are shown in Figure 4. For all values of k_f-bound_, α is within 21% of unity for all the RNA abundance classes and all differential expression ratios examined. In general, the assay is more accurate for rare and intermediate species (error in α within 3% over the entire range of k_f-bound _tested) as compared to abundant species and for lower differential expression ratios compared to higher ratios. For the abundant species, as the forward rate constant for hybridization between mobile and bound species (k_f-bound_) is reduced, the accuracy of the assay decreases. The model predicts that the highest error (a ~1.21) will be observed for abundant species with high differential expression ratios.

The simulation results presented above illustrate that the accuracy index α is most sensitive to the rate constant of the forward reaction for hybridization between mobile and bound species (k_f-bound_) and the deviation of α from unity is highest for abundant species in the sample. The ratio determined in the hybridization assay is . Ideally, this should reflect the true ratio . The ratio [SB']/ [RB'] is always close to S_0_/R_0 _since both S and R hybridize with the same species: R' in solution and B' on the surface. This is because in our analysis, we assume that S and R have identical reaction kinetics, they are thus both stoichiometrically and kinetically indistinguishable from each other. Therefore, the deviation in [RB']/ [R'B] from R_0_/R_0_' (= 1) governs the deviation in γ from the true ratio. Since the reaction is irreversible, for [R'B] to be equal to [RB'], the product [R'] [B] should be equal to [R] [B']. Let ε_S _be the amount of S reacted with R' and ε_R _be the amount of R reacted with R'. Since the stoichiometric ratios for reactions of S with R' and R with R' are both 1, R' =  -ε_R _- ε_S _and R = R_0 _- ε_R_. Under conditions where S_0 _is low (for rare and intermediate species), ε_S _is relatively small and R ≈ R'. However, for abundant species, ε_S _is significant and as a result, R/R' is greater than R_0_/. Also, since

S+B' → SB'

R+B' → RB'

R'+B → R'B

B' gets consumed more than B. Again, for rare and intermediate abundance species, this difference in B and B' is insignificant since the bound strands are in excess and hence the assay works very well for rare and intermediate species. The difference between B and B' is significant for abundant species (S_0 _is large) for which B/B' is greater than B_0_/B_0_'. The exact deviation in B/B' and R/R' depends on the rate constants for hybridization and hence it is not surprising that the rate constants have a major effect on hybridization results. This is illustrated by values for B, B', R and R' for S_0 _= 20 pM, R_0 _=  = 1 pM and k_f-bound _= 10^6 ^M^-1 ^s^-1 ^and 10^4 ^M^-1 ^s^-1^, at the end of 24 hours of hybridization time, presented in Table 3. The products [R] [B'], [R'] [B] and ratios [B]/ [B'] and [R]/ [R'] are also calculated. Thus, for the larger value of k_f-bound_, [R'] [B] ≈ [R] [B'], which is not the case for the lower values of k_f-bound_. This means that factors, such as mixing conditions in the sample chamber, which affect reaction kinetics, will affect the accuracy of the assay.

**Table 3:** An illustration of concentrations of different species at a hybridization time of 24 hours. S_0 _= 10 pM, R_0 _=  = 1 pM

**k_f-bound _M^-1 ^s^-1^**	**[B] pM**	**[B'] pM**	**[R] pM**	**[R'] pM**	**[R] [B'] pM**^2^	**[R'] [B] pM**^2^	**[B]/ [B']**	**[R]/ [R']**
10^6^	9.6 × 10^5^	4.6 × 10^5^	0.63	0.27	2.9 × 10^5^	2.5 × 10^5^	2.09	2.33
10^4^	1.0 × 10^6^	9.9 × 10^5^	0.94	0.40	9.3 × 10^5^	4.0 × 10^5^	1.01	2.35

One implication of the above discussion is that if single stranded species are used for immobilization (only B' immobilized, [B] = 0), as are used in spotted oligo-arrays, the assay will be robust to a wide range of hybridization conditions. This is also seen from our simulations where for all the conditions discussed in this paper, α is equal to one if only sense strands are immobilized on the microarray slide, a situation encountered during the use of oligonucleotide spotted arrays.

## Conclusion

The use of genomic DNA as a reference is useful to assess the expression levels of a large array of genes among different samples. We have developed a kinetic model to predict the effect of using genomic DNA in the microarray assay under a wide range of conditions. The model predicts that the assay can accurately estimate the relative concentrations for a wide range of initial cDNA concentrations and ratios from hybridizations using genomic DNA as a reference will correspond to true ratios under all conditions if single stranded oligonucleotide microarrays are used.

The model also serves as a useful tool to predict the performance of such assays under varying conditions that are otherwise difficult to carry out experimentally. Despite a number of publications on its application, the use of genomic DNA as a reference for microarray assay is still not wide spread. We carried out this study to verify on a theoretical basis the validity of this approach and the results are indeed reassuring. We expect that the use of genomic DNA as reference will accelerate especially for comparative transcriptome analysis involving a wide range of samples from different sources.

## Methods

### Genomic DNA extraction

*Streptomyces coelicolor *A3(2) M145 spores were inoculated into complex media YEME. On reaching stationary phase, mycelia were harvested by centrifugation at 4000 × g and were used to isolate genomic DNA using the Kirby Mix method [24].

### RNA extraction

Two RNA samples were isolated from *S. coelicolor *mycelia obtained from liquid culture at early (Sample 1) and late (Sample 2) growth stages. Mycelia samples were ground in a mortar in the presence of liquid nitrogen and then lysed by addition of RLT buffer from the RNeasy Mini Kit (Qiagen Inc., Valencia, CA). Total RNA was then isolated from the lysate using the RNeasy Mini Kit according to the manufacturer's protocol.

### Microarray hybridization

*S. coelicolor *microarray containing duplicate spots representing 90% of the genes in the genome was used for hybridizations to compare RNA from Samples 1 and 2. The construction of the microarray is described on our website at .

#### cDNA: cDNA hybridization

10 μg of total RNA was used for each sample as starting material. Total RNA was reverse transcribed into cDNA incorporating aa-dUTP (Ambion, Austin, TX) and then labeled with Cy3 (Amersham Biosciences, Piscataway, NJ) or Alexa647 (Invitrogen, Carlsbad, CA).

Data presented here is an average from four replicate hybridizations. For two hybridizations, Sample 1 was labeled with Cy3 and Sample 2 with Alexa647, while for the other two hybridizations, the dyes were reversed with Sample 1 labeled with Alexa647 and Sample 2 with Cy3.

#### Genomic DNA hybridization

Hybridization with genomic DNA (gDNA) was carried out using 10 μg total RNA and 200 ng genomic DNA. Genomic DNA was nebulized to the length range of 500 bp to 1 kb. A nebulizer containing 2 ml of buffered genomic DNA solution (approximately 1 mg) containing 40% glycerol was placed in an ice-bath and was subjected to nitrogen gas at a pressure of 25 psi for 3 minutes. The resulted DNA fragments were purified by ethanol precipitation and were resuspended to a concentration of about 1 μg/μl. The fragmented genomic DNA was then labeled with Cy3 dye using *Label *IT^® ^Cy™3 Labeling Kit (Mirus, Madison, WI). The labeling reaction consisted of 20% *Label *IT Reagent and 1 μg genomic DNA in 7 μl reaction volume. The reaction was incubated at 37°C for 3 hours and the labeled genomic DNA was purified with MinElute PCR purification kit (Qiagen Inc., Valencia, CA) as per the manufacturer's instructions. For hybridization with genomic DNA, cDNA was labeled with Alexa647 (Invitrogen, Carlsbad, CA). Four replicate hybridizations were performed and ratios obtained from the four hybridizations were averaged as described below.

All hybridizations were carried out at 50°C for 16 hours. Details of all protocols are available on our website at  or are available as supplimentary material (see Additional file 2). Microarray slides were scanned after hybridization and washing using ScanArray (PerkinElmer, Boston, MA) and the images were quantified using GenePix Pro 5.1 (Axon Instruments, Union City, CA). The median intensity for each spot was used for further analysis.

#### Data analysis

Four replicate hybridizations were performed for each experiment with the following pairs of fluorescently labeled samples: cDNA_1_-gDNA, cDNA_2_-gDNA and cDNA_1_-cDNA_2_. Thus, for each experiment 8 replicate data points were obtained for each gene. The median intensity of pixels within a spot was used for analysis. Bad spots were filtered out based on the following criteria: 1) Spots flagged based on visual inspection during image analysis; 2) Spots with diameter was less than 70 μm; 3) Spots where the intensity of both channels was less than 200. The remaining 'good' spots were then normalized. LOWESS, a non-linear normalization algorithm from the commercial software GeneSpring (Silicon Genetics, Redwood City, CA), was used for cDNA:cDNA hybridization and linear normalization was used for cDNA:gDNA hybridization. For the linear normalization, we linearly scale the intensities of all the spots within each channel so that the sum of intensities of all spots in one channel is equal to 40,000,000. For all three experiments, after normalization, the average and standard deviation (SD) of the log_2 _transformed ratio for all the replicate spots for each gene was calculated. Spots outside the range [mean - 1.5 SD, mean + 1.5 SD] were considered outliers and therefore discarded. The average of the log_2 _transformed ratios for the remaining spots was calculated.

## Authors' contributions

MG developed the model, carried out simulations, analyzed results and drafted the manuscript. WL carried out the microarray hybridization experiments. CG participated in model development and analysis of results. VK supervised the project. WSH conceived of the study, participated in its design and analysis and supervised the project. All authors participated in writing the manuscript and approved the final manuscript.

## Supplementary Material

Additional File 1 Microarray data in a tab-delimited text format with arbitrary gene identifiers for the *S. coelicolor *genes. Click here for file

Additional File 2 Details of the microarray hybridization protocols used in PDF format. Click here for file
