# Supplementary material for: An analysis of the use of genomic DNA as a universal reference in two channel DNA microarrays
Source: BMC Genomics. 2005 May 8;6:66. doi: 10.1186/1471-2164-6-66 (PMC1142311; doi:10.1186/1471-2164-6-66)
Supplement: Additional File 2 — Details of the microarray hybridization protocols used in PDF format. [file 1471-2164-6-66-S2.pdf]

Wei-Shou Hu Laboratory
Department of Chemical Engineering
and Materials Science
University of Minnesota

Design of primers for *Streptomyces coelicolor* whole genomic cDNA microarray

Primers were designed with **Primer3** using **Primer_Design_Project** from Arkady Khodursky's lab in UMN. Primers were designed in a few rounds to avoid the following potential situations: i) primer pairs bind to non-specific sequences and can also PCR amplify a fragment with reasonable length; ii) PCR products have homology to other genes. The following chart shows the process of our primer design. Most of the designed primers give probes about 500 bp long and most primers have melting temperatures about 62 °C.

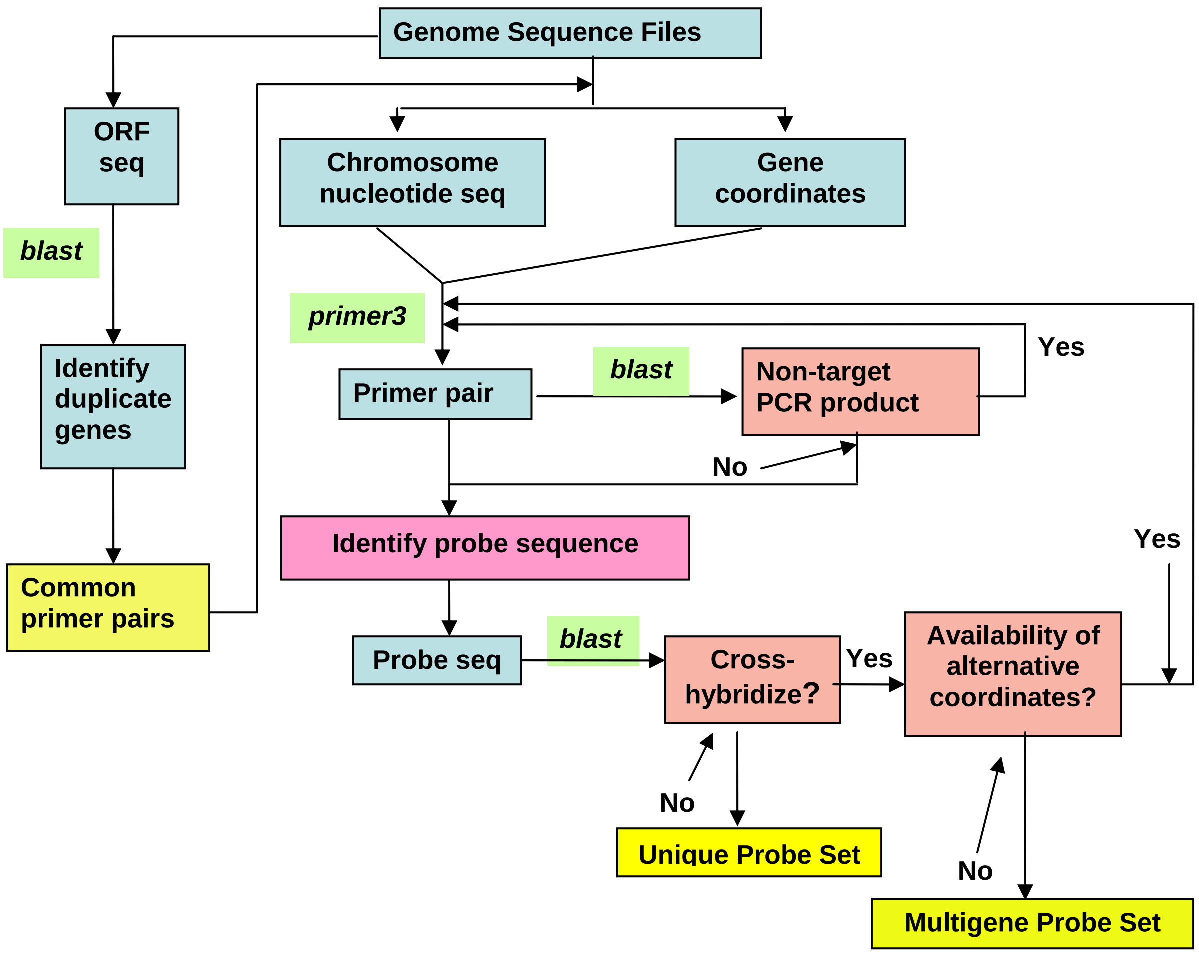

Wei-Shou Hu Laboratory
Department of Chemical Engineering
and Materials Science
University of Minnesota

Protocol for PCR *Streptomyces coelicolor* Genome:

Materials

1. Master Mix -10ml (for 100 PCR rxns)

Reagent	Amount	Final conc per 100 µl PCR rxn
10 x Takara EX Taq Buffer without $MgCl_2$	1 ml	1 x
$MgCl_2$ (1 M)	10 µl	1 mM
genomic DNA (purified using Kirby Mixture)	1 µg	10 ng/100 µl
dCTP (100 mM)	20 µl	200 µM
dGTP (100 mM)	20 µl	200 µM
dATP (100 mM)	8.6 µl	86 µM
dTTP (100 mM)	8.6 µl	86 µM
DMSO	100 µl	1 µl/100 µl
glyerol	800 µl	8 µl/100 µl
Taq	500 units	5 µl/100 µl
Add balance of Milli-Q H_2O to 100 µl		

2. 10X PCR Buffer Composition

Tris (pH 8.3)	1 M
KCl	0.5 M
$MgCl_2$	10 mM

Equipments

1. Thermocycler
2. Centrifuge with a plate rotor

Procedure

1. Make master mix for more than 1 plate reactions (96 rxns) containing the reagents listed above, except primers
2. Add 98 µl of master mix into each well of 96-well thermal PCR plates using a 12-channel pipette
3. Transfer to each well 2 µl of 25 µM primers from Costar 96-well primer stock plates with a 12-channel pipette
4. Use the 12-channel pipette to mix the reaction mixture, seal the plate
5. Centrifuge the plates
6. Place the reaction plate into PCR machines

PCR profile

1. Initial Denature: 96° C for 5 min.
2. 40 cycles:

   94° C for 20 s
   62° C for 20 s
   72° C for 20 s
3. Final Extension: 72°C for 5 minutes
4. 15°C soak

Special Notes

Some primers failed to generate PCR products using Takara EX taq buffer (see XXX for a list of these primers). For the ones that didn't work, use the following PCR buffer in place of Takara EX Taq buffer.

Wei-Shou Hu Laboratory
Department of Chemical Engineering
and Materials Science
University of Minnesota

Protocols for PCR Clean Up

Materials

- 3 M NaOAc pH 5.2
- IPA
- 70% Ethanol (made from 95% Ethanol)
- Spotting buffer (3 X SSC + 1.5 M Betaine)
- Costar 96-well v-bottom polypropylene plates
- Greiner 384-well v-bottom polypropylene plates

Equipments

- Centrifuge with plate rotor of maximum speed > 4500 g

Precipitation Method

1. After PCR reaction, add 10 µl 3 M NaOAc pH 5.2 to each well
2. Transfer reactions to a Costar 96-well V-bottom Polypropylene Plate. To save on pipette tips, first add 100 µl room temperature IPA to Costar plates and then transfer PCR + NaOAc pH 5.2 to IPA. Mix well with pipette
3. Transfer PCR reaction mixture (to which NaOAc has been added)
4. Spin plates at 4700 rpm for at least 2 hrs (at most 6 plates a time)
5. Quickly discard the supernatant right after the spinnin
6. Add 100 µl of ice cold 70% EtOH
7. Centrifuge the plates for half an hour at 4700 rpm
8. Discard the supernatant
9. Let plates air dry or dry in the speed vacuum with the plate rotor.
10. Resuspend the DNA pellets in spotting buffer for at least 10 hrs and save in -20 °C. Make sure the plates are sealed well. The PCR products can be directly transferred to Greiner 384 Well Polypropylene Plates at any time.

Wei-Shou Hu Laboratory
Department of Chemical Engineering
and Materials Science
University of Minnesota

Filtration Method Using Millipore Filter Plates

Materials

- Montage PCRµ96 Filter Plate (Millipore)
- TE buffer
- Spotting buffer (3 X SSC + 1.5 M Betaine)
- Greiner 384-well v-bottom polypropylene plates

Equipments

- MultiScreen® Resist Vacuum Manifold (Millipore)
- Vacuum pump
- Shaker

1. Add 5-µL TE buffer to each well of the 96-well plate containing the PCR product to be purified.
2. Transfer the PCR product and buffer to a Millipore Montage $PCR_{\mu 96}$ filter plate.
3. Place the filter plate on the vacuum holder. Turn on vacuum pump and adjust pressure to 20-inHg.
4. Allow the PCR product to filter until the filter is dry and shiny looking. (Approximately 12-18 minutes).
5. Place an additional 50-µL TE buffer to each well of the filter plate, and allow the vacuum to run until the filter is again dry and shiny looking.
6. Remove filter from vacuum system, and blot the bottom of the filter dry with a paper towel.
7. Add spotting buffer to each well of the filter plate, and place the filter on a shaker for 20-30 minutes.
8. Transfer the purified PCR product directly to Greiner 384 Well Polypropylene Plates. Seal the plate well store at -20 °C.

Note: Filter Plates are good for PCR product more than 100 bp long.

Wei-Shou Hu Laboratory
Department of Chemical Engineering
and Materials Science
University of Minnesota

Protocol for preparing poly-l-lysine slides for microarrays

Developed by the DeRisi lab, UCSF

Modified by the Hu lab, U of MN

Basics

The following protocol is for coating 30 slides with poly-l-lysine.

1. -DO NOT use powdered gloves at any time during this protocol.
2. -To avoid dust, try to keep slides covered or submerged in solution at all times.
3. -Once coated, slides are usually stable for 4 months.

Materials

1. Poly-L-lysine (Sigma)
2. Sodium Hydroxide pellets
3. 95% Ethanol
4. Filtered PBS Solution

Equipments

1. Slide racks
2. Glass slide dishes
3. Plastic slide dishes
4. Centrifuge with plate rotor
5. Vacuum oven

Procedure

1. Prepare wash solution for 30 slides:

NaOH pellets	33 g
95% EtOH	200 ml
H_2O	133 ml

2. Mix wash solution until NaOH pellets are completely dissolved.
3. Rinse slide dishes completely with Milli-Q H_2O. Add one rack of GoldSeal Microslides per slide dish. Pour wash solution over slides and cover.
4. Shake slides gently for 2 hours in wash solution. Although the slides come "prewashed" there are still significant amounts of oils and debris. This basic wash is necessary to completely clean the slides before applying the poly-l-lysine.
5. Rinse slides with several liters of Milli-Q H_2O. Fill a large container with H_2O so that you can vigorously dump the rinse over the slides. Let the slides sit in the water while you prepare the poly-l-lysine solution.
6. Prepare poly-l-lysine solution (**use only plastic-ware when preparing poly-l-lysine) for 30 slides:

Milli-Q H_2O	250 ml
1X PBS (filtered)	35 ml
poly-l-lysine	60 ml

7. Mix ingredients on stir plate in plastic beaker in order listed above.
8. Before adding poly-l-lysine, dump H_2O from slides. Immediately after adding poly-l-lysine, pour solution over slides.
9. Let slides shake gently in poly-l-lysine for 30 minutes.
10. Rinse slides again with Milli-Q H_2O in same fashion as described above.
11. Immediately spin slides in a tabletop centrifuge at 500 rpm until dry (approx. 5 min).
12. To ensure slides are completely dry, place racks in 50 °C vacuum oven for at least 5 min. If a vacuum oven is not available, place the slides in a standard oven for a longer amount of time.
13. Store slides in a clean plastic slide box. Slides MUST be stored for at LEAST 14 days before spotting DNA. We do not recommend using slides that are more than 4 months old as degradation of poly-l-lysine has been observed.

Note: Poly-l-lysine solution can be reused if you expect to prepare a large volume of slides. To do so, filter lysine solution after step 9 and store at 4 °C in a plastic container. When ready to use, distribute solution to slide dishes and add an additional 2-3 ml of new poly-l-lysine to each dish. This should be repeated a maximum of 6 times.

Wei-Shou Hu Laboratory
Department of Chemical Engineering
and Materials Science
University of Minnesota

Printing

Biorobotics MicroGrid II with MicroSpot 2500 pins is used to print arrays in the two core facilities in UMN. To prevent carry-over contaminations, pins are washed between different source visits: 2 seconds in each of the two re-circulating wash tanks, 2 seconds flush and 30 seconds vacuum in the Main Wash Station; and the whole cycle repeats once more. Humidity is controlled at 50% during printing.

Array spotting is carried out in either of the two core facilities in University of Minnesota by Archana Deshpande in BIPL/CGAC or Todd Markowski in AGAC. Both core facilities use Biorobotics MicroGrid II with MicroSpot 2500 pins for printing. To prevent carry-over contaminations, pins are washed between different source visits: 2 seconds in each of the two re-circulating wash tanks, 2 seconds flush and 30 seconds vacuum in the Main Wash Station; and the whole cycle repeats once more. Humidity is controlled at 50% during printing.

Wei-Shou Hu Laboratory
Department of Chemical Engineering
and Materials Science
University of Minnesota

Protocol for post processing microarrays

Materials

1. Succinic anhydride (stored in vacuum desiccator)
2. Blocking solution – 1,2-dichloroethane and N-methylimidazole (make fresh – see details in the protocol)
3. 95% Ethanol

Equipments:

1. Oven
2. Slide racks and slide dishes
3. UV-crosslinker
4. Humid Chamber
5. Stir Plate
6. Centrifuge with plate rotor

Before beginning:

1. Set the oven to 80 °C
2. Boil water in microwave, enough to cover slide rack
3. Use powder free gloves throughout protocol.
4. DO NOT splash any solutions on array during hydration and heat fixing

Procedure: Hydration/fixing

Each rack holds maximum 30 slides. Ideally each time one can process 20-30 slides

1. On the back of slide, etch two lines above and below center of array to designate array area after processing. Post processing causes the printed area to become invisible.
2. Pour 100 ml 0.5 X SSC into humid chamber; warm up to 37-40 °C on a slide warmer at medium setting or use a heat block
3. Set slide array side down and observe spots until proper hydration is achieved (1 min to 2 min).
4. Place slides on the rack.

5. Bake the slides in 80 °C oven for 60-90 min.
6. UV cross-link the slides with 65 mJ - set at 650 (x 100 µJ).

Procedure: surface blocking

(Adapted from *Diehl et. al., 2001 NAR 29: e38*)

1. Make sure that the slide dish is completely dry, water free. Otherwise it should be oven dried.
2. Dissolve 1.75 g succinic anhydride in 350 ml anhydrous 1,2-dichloroethane in a clean, dry slide dish using a stirrer bar.
3. Immediately after the last flake of the succinic anhydride dissolves (~ 5 min), add the 4.4 ml of N-methylimidazole.
4. Dip the slides in the Blocking Solution, gently up and down several times, then incubate for 1 hr on an orbital shaker (or magnetic stirrer with constant agitation).
5. Wash vigorously in Milli-Q H_2O a few times until slides look clean and not slimy.
6. Optional: Plunge the slides up and down gently a few times into 95 °C Milli-Q H_2O and let it sit for 2 min. This step will denature the double stranded DNA. Since our printing buffer contains 1.5 M betaine, the PCR product could be already denatured. Skipping this step doesn't reduce hybridization signals.
7. Dip the slides into 95% ethanol for 2 minutes, again up and down a few times and then let it sit. Transfer slides into a centrifuge and spin for 5 minutes at 500 rpm.
8. Put the slides back to the same Blocking Solution used in step 4. Repeat step 4, 5, and 7.
9. Arrays may be used immediately. If not, they should be stored in a desiccator for future use.

Wei-Shou Hu Laboratory
Department of Chemical Engineering
and Materials Science
University of Minnesota

Protocols for gDNA nebulization

Modified by the Hu Lab, UMN from a online protocol at
http://www.genome.ou.edu/protocol_book/protocol_partII.html#II

Materials

- Nebulizer (IPI Medical Products Inc., Chicago, IL)
- 3 M NaOAc pH 5.2
- Ethanol
- Glycerol
- 10 X TM buffer

Tris Buffer, pH 8.0	500 mM
$MgCl_2$	150 mM

Equipments

- Nitrogen gas tank
- Microcentrifuge

Protcol

1. Modify a nebulizer by removing the plastic cylinder drip ring, cutting off the outer rim of the cylinder, inverting it and placing it back into the nebulizer. Seal the large hole in the top cover with a cork and connect a tubing (which eventually should be connected to a nitrogen gas tank) to the smaller hole.
2. Prepare the following DNA sample (total of 2 ml) and place in the nebulizer cup:

gDNA	1 mg
10 X TM buffer	200 µl
Glycerol	800 µl
Milli-Q H_2O	balance the rest volume for total of 2 ml

3. Close the top cover and connect the tubing from the cover to a liquid nitrogen tank
4. Nebulize the DNA sample in an ice-water bath at 25 psi for 3 minutes
5. Distribute the sample into four 1.5 ml microcentrifuge tubes and add 1/10 volume of 3M NaAc and 2 volumes of ethanol. Centrifuge at 13,000 rpm for 5 min and wash with 70% ethanol, air dry the pellets and resuspend in Milli-Q H_2O to a concentration of about 1 µg/µl.

6. Run electrophoresis gel to double check the size distributation (range of 500 bp to 1.5 kp is fine)

Wei-Shou Hu Laboratory
Department of Chemical Engineering
and Materials Science
University of Minnesota

RNA isolation protocol for *S. coelicolor*

Materials

1. Hydrogen Peroxide
2. RNeasy Mini Kit (Qiagen)
3. RNase-free DNase Set (Qiagen)
4. Liquid nitrogen

Equipments

1. Mortars and pestles
2. Microcentrifuge

Procedure

I. Preparation of mortar and pestle

1. Immerse the mortar and pestle in 3% H_2O_2 for at least half an hour.
2. Rinse them with RNase-free water (double autoclaved Milli-Q H_2O can be used).
3. Wrap a pair of mortar and pestle in a clean aluminum foil, and bake overnight at 180 °C.
4. Cool it down to above supernatent room temperature and keep it in – 80 °C for half an hour.

II. Cell disruption and lysis

1. Pour liquid nitrogen into the mortar.
2. Take out mycelia sample from – 80 °C freezer and use pipette tip to push out the pellet from the bottom of tube into the mortar (Don't warm up the sample too much. Once the frozen pellet melts, it's very hard to take all sample out into mortar)
3. Grind the pellet completely (Add more liquid nitrogen, if necessary)
4. Add 500 µl of buffer RLT to the sample. Wait until the buffer melts, then mix everything again with pestle
5. Transfer the lysate into a new microcentrifuge tube and vortex thoroughly.

III. RNA isolation

1. Spin the sample at 12,000 rpm for 1 min and transfer the supernatant into a new tube.

2. Add 250 µl ethanol to the lysate. Mix thoroughly by pipetting. DO NOT centrifuge.
3. Apply the mixture (~ 700 µl), including any precipitate, into an RNeasy mini column placed in 2 ml collection tube. Centrifuge for 15 sec at >10,000 rpm. Discard the flow-through.
4. Add 350 µl Buffer RW1 into the column, and centrifuge for 15 sec at 10,000 rpm to wash. Discard the flow-through.
5. Add 10 µl DNase I stock solution (RNeasy mini handbook, pp. 99) to 70 µl Buffer RDD. Mix gently by inverting the tube.
6. Pipet the DNase I incubation mix (80 µl) directly onto the RNeasy silica-gel membrane and place on the benchtop (20-30 °C) for 15 min.
7. Pipet 350 µl Buffer RW1 into the column, and centrifuge for 15 sec at >10,000 rpm. Discard flow through.
8. Add 700 µl Buffer RW1 to the column. Centrifuge for 15 sec at >10,000 rpm to wash the column. Discard the flow-through and the collection tube.
9. Transfer the RNeasy column into a new 2 ml collection tube. Pipet 500 µl Buffer RPE onto the column. Centrifuge for 15 sec at >10,000 rpm to wash the column. Discard the flow-through.
10. Add another 500 ul Buffer RPE to the column. Centrifuge for 15 sec at >10,000 rpm to wash the column. Discard the flow-through.
11. Centrifuge for another 2 min at >10,000 rpm to dry the RNeasy silica-gel membrane.
12. To elute, transfer the column to a new 1.5 ml centrifuge tube. Pipet 30-50 µl RNase-free water directly onto the column membrane. Let it sit in RT for 1 min and centrifuge for 1 min at >10,000 rpm to elute.
13. Repeat the elution step (step 12) with more RNase free water for higher yield or using the flow-through from step 12 for higher concentrations.
14. Store at –80 °C.

Wei-Shou Hu Laboratory
Department of Chemical Engineering
and Materials Science
University of Minnesota

Protocol for *Streptomyces coelicolor* Microarray Hybridization (cDNA:cDNA)

Materials

1. MinElute PCR Purification Kit (Qiagen)
2. SuperScript II (Invitrogen)
3. Random hexamer - pdN6 (IDT)
4. Alexa Fluor® 647 reactive dye and Alexa Fluor® 555 reactive dye (Invitrogen)
5. 0.1 M $NaHCO_3$ Buffer (pH 9.0)
6. 4 M hydroxylamine
7. 3 M NaOAc; Ethanol
8. Cover Glass – 24 mm X 60 mm
9. dNTP mix (100 µl)

10 mM dGTP	10 µl
100 mM dCTP	10 µl
100 mM dATP	4 µl
100mMdTTP	0.4 µl
10mM aa-dUTP	36 µl
RNase-free H_2O	39.6 µl

10. RT Cocktail (14µl per rxn)

5 x RT buffer	6 µl
DTT	3 µl
dNTP	3 µl
RNase-free H_2O	2 µl

11. 20 X SSC
12. 10% SDS
13. Salmon sperm DNA

Equipments

1. Microcentrifuge
2. Thermocycler
3. Slide racks and slide dishes
4. Hybridization Chamber

Wei-Shou Hu Laboratory
Department of Chemical Engineering
and Materials Science
University of Minnesota

Procedure

I. RT Reaction

1. Mix in a PCR tube in total volume of 15 µl:
   total RNA 10 µg
   pdN6 9 µg
2. Incubate the mixture at 70 °C for 10 min
3. Chill on ice for 5 min
4. Add 14 µl RT cocktail
5. Add 1 µl Superscript II and mix the tube
6. Start RT reaction in a thermocycler using the following program:

   25 °C - 10 min

   37 °C - 5 min

   42 °C - 1 hr 45 min

II. Hydrolysis

1. Add 10 µl 0.5 M EDTA and 10 µl 1 N NaOH
2. Incubate at 65 °C for 15 min
3. Add 25 µl 1 M Tris-HCl pH 7.5, mix well
4. Samples may be stored at 4°C overnight at this point

III. Clean up with isopropanol precipitation

1. Transfer samples from PCR tube into a new microcentrifuge tube
2. Add 1/10 volume 3 M NaOAc and 3 volume ethanol, mix well
3. Spin for 5 min at 13,000 rpm
4. Wash with 70% ethanol
5. Air dry or speed vacuum dry the pellet
6. Samples may be stored at -20°C forever

Note: An alternative method to clean up is using Microcon 30 concentrator.

IV. Coupling

Note: Dyes are light sensitive. Avoid overhead fluorescent lighting as much as possible.

1. Resuspend cDNA pellet in 9 µl 0.1M $NaHCO_3$ Buffer (pH 9.0). Let it sit for 10-15 min at room temperature to ensure resuspension

2. In the mean time, take out one tube of each Alexa dye, let it sit for 10 min at RT before resuspending with 8 µl DMSO
3. Use Alexa 555 for one sample and Alexa 647 for the other.
4. Transfer 2 µl of the dye into the resuspended cDNA and mix by pipetting (The rest of dye can be saved in -20°C and used for a week)
5. Incubate for 1 hr at room temperature in the dark

V. Quenching and cleanup

Before combining two samples for hybridizations, the reactions must be quenched to prevent cross-coupling.

1. Add 4.5 µl 4 M hydroxylamine. Let reaction incubate for 15 min at room temperature in dark.

To remove unincorporated/quenched Alexa dye proceed with MinElute PCR Purification Kit

2. Combine two reactions in one microcentrifuge tube
3. Add 70 µl water
4. Add 500 µl Buffer PB
5. Apply to MinElute column and spin at 13,000 rpm in for 30-60 sec
6. Discard flow-thru
7. Add 500 µl Buffer PE and spin 30-60 sec
8. Duiscard flow-thru and repeat.
9. Discard flow-thru and centrifuge for 1 min at high speed to dry column
10. Transfer to a fresh centrifuge tube
11. Add 10 µl Buffer EB to center of filter and let sit 1 min at room temperature
12. Centrifuge at 13,000 rpm for 1 min
13. Dry down the eluate – labeled cDNA in speed vacuum

VI. Hybridization prepare

1. Add the following reagents into the tube containing dry labeled cDNA and mix with a pipette:

Formamide	20 µl
20 X SSC	8 µl
4% SDS	1 µl
Salmon sperm DNA	3 µl
Milli-Q H_2O	8 µl

2. Denature the cDNA probe for 2 min at 98 °C and cool down to 50 °C

VII. Array hybridization

1. Put a 20 µl drop of 3XSSC at each end of the hybridization chamber. This is to ensure a constant humidity in the chamber during hybridizations. If the 3XSSC is not applied, the array will dry out.
2. Take out one clean cover glass from the box. Put the probe onto one edge of the array in a drop (avoiding bubbles). The cover glass should be placed on the array starting at the edge next to the probe while you slowly lower the other end onto the slide with a forceps.
3. Tightly screw down hybridization chamber lid and carefully place chamber in a 50 °C water bath. Take caution to keep array completely flat during transfer and hybridization.
4. Allow hybridization to run for at least 8 hours but no more than 24 hours.

VIII. Array Wash

1. Prepare wash solutions in glass slide dishes, with each dish having its own rack

**Wash Soln. I**	**Wash Soln. II**
340 ml Milli-Q H_2O	350 ml Milli-Q H_2O
10 ml 20 X SSC	1 ml 20 X SSC
1 ml 10% SDS	

2. Carefully remove array from water bath
3. Dry array with paper towels and attempt to "wick" any water away from chamber seams
4. Unscrew chamber and remove array
5. Once submerged tilt array and gently dump off cover glass. It may be necessary to lightly swish array under solution to dislodge the slip
6. Once the cover glass is off and lying on bottom of slide dish, put array in rack from water bath. **DO NOT** allow slides to dry out at any point during the washes. When all the slides are in Wash I, plunge the rack up and down for at least 1 min
7. Individually transfer slides to slide dish containing Wash II, do not transfer entire slide rack as this will cause too much SDS carryover. Plunge for at least 1 min again
8. Dry array in room temperature table top centrifuge at 500 rpm for 5 min
9. Try to scan array within hours of washing as the dyes are instable and degrade differentially

Wei-Shou Hu Laboratory
Department of Chemical Engineering
and Materials Science
University of Minnesota

Protocol for Microarray Hybridization (gDNA:cDNA)

Materials

1. MinElute PCR Purification Kit (Qiagen)
2. *Label* IT® Nucleic Acid Cy™3 Labeling Kit (Mirus)
3. Cover Glass – 24 mm X 60 mm
4. SuperScript II (Invitrogen)
5. Random Hexamer - pdN6
6. 0.1 M $NaHCO_3$ Buffer (pH 9.0)
7. 4 M hydroxylamine
8. 3 M NaOAc; Ethanol
9. Alexa Fluor® 647 reactive dye (Invitrogen)
10. dNTP mix (100 µl)

100 mM dGTP	10 µl
100 mM dCTP	10 µl
100 mM dATP	4 µl
100 mM dTTP	0.4 µl
10 mM aa-dUTP	36 µl
RNase-free H_2O	39.6 µl

11. RT cocktail, 14 µm per rxn

5 X RT buffer	6 µl
DTT	3 µl
dNTP mix	3 µl
RNase-free H_2O	2 µl

12. 20 x SSC
13. 10% SDS
14. Salmon sperm DNA

Wei-Shou Hu Laboratory
Department of Chemical Engineering
and Materials Science
University of Minnesota

Equipments

1. Microcentrifuge
2. Thermocycler
3. Slide racks and slide dishes
4. Hybridization Chamber

Procedure

I. Preparation of gDNA probe

1. Sonicate or nebulize genomic DNA to about 500 bp to 1 kb range
2. Label gDNA with Mirus labeling kit
   Note: If labeling more than 1 µg gDNA, scale up the following volumes proportionally.
   a) Take out the reconstituted Label IT Reagent from the freezer and let it sit at RT for 10 min before open the cap (to prevent moisture from entering the tube)
   b) Mix in a tube of total volume 7 µl:

10 x Labeling Buffer A	0.7 µl
1 µg/µl fragmented gDNA	1.0 µl
Label IT Reagent	1.4 µl
Milli-Q H_2O	3.9 µl

   c) Incubate at 37 °C for 3 hrs
3. Clean up with Qiagen MinElute PCR purification kit (one column for maximum 5 µg labeled gDNA)
   a) Add 70 µl water
   b) Add 500 µl Buffer PB
   c) Apply to a MinElute column and centrifuge at 13,000 rpm in for 30-60 sec
   d) Discard flow-thru
   e) Add 500 µl Buffer PE and centrifuge 30-60 sec
   f) Discard flow-thru and repeat.
   g) Discard flow-thru and spin for 1 min at high speed to dry column
   h) Transfer to fresh microcentrifuge tube
   i) Add 10 µl (per 1 µg gDNA) EB Buffer to the center of filter and let sit 2 min at room temperature
   j) Spin at 13,000 rpm for 1 min

4. Denature gDNA probe
   a) Add 1/10 volume of Mirus Denature Buffer D1
   b) Mix and incubate at room temperature for 5 min
   c) Add 1/10 volume of Mirus Neutralization Buffer N1, mix and incubate on ice for at least 5 min
   d) If not to be used immediately, keep in -20 °C (can be thawed and used directly); whenever the labeled gDNA is taken out for use again, it should be kept on ice.

II. Preparation of cDNA probe

i. RT Reaction

1. Mix in a PCR tube in total volume of 15 µl:
   total RNA 10 µg
   pdN6 9 µg
2. Incubate the mixture at 70 °C for 10 min
3. Chill on ice for 5 min
4. Add 14 µl RT cocktail
5. Add 1 µl Superscript II and mix everything
6. Start RT reaction in a thermocycler using the following program:
   25 °C - 10 min
   37 °C - 5 min
   42 °C - 1 hr 45 min

ii. Hydrolysis

1. Add 10 µl 0.5 M EDTA and 10 µl 1 N NaOH into the above reaction
2. Incubate at 65 °C for 15 min
3. Add 25 µl 1 M Tris-HCl pH 7.5, mix well
4. Samples may be stored at 4 °C overnight at this point
5. Transfer samples from the PCR tube into a new microcentrifuge tube
6. Add 1/10 volume 3M NaOAc and 3 volume ethanol, mix well
7. Spin for 5 min at 13,000 rpm
8. Wash with 70% ethanol
9. Air dry or speed vacuum dry the pellet
10. Samples may be stored at -20 °C for ever

Note: An alternative method to clean up is using Microcon 30 concentrator.

iii. Coupling

NOTE: Dyes are light sensitive. Avoid overhead fluorescent lighting as much as possible.

1. Resuspend cDNA pellet in 9 µl 0.1M $NaHCO_3$ Buffer (pH 9.0). Let it sit for 10-15 min at RT to ensure resuspension
2. In the mean time, take out one tube of Alexa 647 dye, let it sit for 10 min at RT before resuspending with 8 µl DMSO
3. Transfer 2 µl of the dye into the resuspended cDNA and mix by pipetting (The rest of dye can be saved in -20 °C and used for at least a week)
4. Incubate for 1 hr at room temperature in the dark

iv. Quenching and Cleanup

To remove unincorporated Alexa dye proceed with MinElute PCR Purification Kit

1. Add 70 µl Milli-Q H_2O to above coupling mixture
2. Add 500 µl Buffer PB
3. Apply the new mixture to Qia-quick column and spin at 13,000 rpm in for 30-60 sec
4. Dump off flow-thru
5. Add 500 µl Buffer PE to the column and spin for 30-60 sec
6. Dump flow-thru and repeat step 5
7. Dump flow-thru and spin for 1 min at high speed to dry column
8. Transfer the column to a fresh microcentrifuge tube
9. Add 15 µl Buffer EB to the center of filter and let sit for 2 min at room temperature
10. Spin at 13,000 rpm for 1 min
11. Dry down the eluate in speed vacuum

III. Hybridization Prepare

1. Add the following reagents into the microcentrifuge tube containing dry labeled cDNA and mix with the pipette:

Formamide	20 µl
20 X SSC	8 µl
4% SDS	1 µl
Salmon sperm DNA	3 µl
Milli-Q H_2O	6 µl

2. Denature the cDNA probe for 2 min at 98 °C and cool down to 50 °C
3. Add 2 µl labeled gDNA and mix

IV. Array Hybridization

1. Put a 20 µl drop of 3XSSC at each end of the hybridization chamber. This is to ensure a constant humidity in the chamber during hybridizations. If the 3XSSC is not applied, the array will dry out.
2. Take out one clean cover glass from the box. Put the probe onto one edge of the array in a drop (avoiding bubbles). The cover glass should be placed on the array starting at the edge next to the probe while you slowly lower the other end onto the slide with a forceps.
3. Tightly screw down hybridization chamber lid and carefully place chamber in a 50 °C water bath. Take caution to keep array completely flat during transfer and hybridization.
4. Allow hybridization to run for at least 8 hours but no more than 24 hours.

V. Array Wash

1. Prepare wash solutions in glass slide dishes, with each dish having its own rack

**Wash Soln. I**	**Wash Soln. II**
340 ml Milli-Q H_2O	350 ml Milli-Q H_2O
10 ml 20 X SSC	1 ml 20 X SSC
1 ml 10% SDS	

2. Carefully remove array from water bath
3. Dry array with paper towels and attempt to "wick" any water away from chamber seams
4. Unscrew chamber and remove array
5. Once submerged tilt array and gently dump off cover glass. It may be necessary to lightly swish array under solution to dislodge the cover glass
6. Once the cover glass is off and lying on bottom of slide dish, put array in the slide rack. **DO NOT** allow the slide to dry out at any point during the washes. When all the slides are in Wash I, plunge the rack up and down for at least 1 min
7. Individually transfer slides to slide dish containing Wash II, do not transfer entire slide rack as this will cause too much SDS carryover. Plunge for at least 1 min again
8. Dry array in room temperature table top centrifuge at 500 rpm for 5 min
9. Try to scan array within hours of washing as the dyes are instable and degrade differentially
